# Systematic review of randomized clinical trials on the use of hydroxyethyl starch for fluid management in sepsis

**DOI:** 10.1186/1471-227X-8-1

**Published:** 2008-01-24

**Authors:** Christian J Wiedermann

**Affiliations:** 1 Division of Internal Medicine 2, Department of Medicine, Central Hospital of Bolzano, Bolzano, Italy

## Abstract

**Background:**

Patients with sepsis typically require large resuscitation volumes, but the optimal type of fluid remains unclear. The aim of this systematic review was to evaluate current evidence on the effectiveness and safety of hydroxyethyl starch for fluid management in sepsis.

**Methods:**

Computer searches of MEDLINE, EMBASE and the Cochrane Library were performed using search terms that included hydroxyethyl starch; hetastarch; shock, septic; sepsis; randomized controlled trials; and random allocation. Additional methods were examination of reference lists and hand searching. Randomized clinical trials comparing hydroxyethyl starch with other fluids in patients with sepsis were selected. Data were extracted on numbers of patients randomized, specific indication, fluid regimen, follow-up, endpoints, hydroxyethyl starch volume infused and duration of administration, and major study findings.

**Results:**

Twelve randomized trials involving a total of 1062 patients were included. Ten trials (83%) were acute studies with observation periods of 5 days or less, most frequently assessing cardiorespiratory and hemodynamic variables. Two trials were designed as outcome studies with follow-up for 34 and 90 days, respectively. Hydroxyethyl starch increased the incidence of acute renal failure compared both with gelatin (odds ratio, 2.57; 95% confidence interval, 1.13–5.83) and crystalloid (odds ratio, 1.81; 95% confidence interval, 1.22–2.71). In the largest and most recent trial a trend was observed toward increased overall mortality among hydroxyethyl starch recipients (odds ratio, 1.35; 95% confidence interval, 0.94–1.95), and mortality was higher (p < 0.001) in patients receiving > 22 mL·kg^-1 ^hydroxyethyl starch per day than lower doses.

**Conclusion:**

Hydroxyethyl starch increases the risk of acute renal failure among patients with sepsis and may also reduce the probability of survival. While the evidence reviewed cannot necessarily be applied to other clinical indications, hydroxyethyl starch should be avoided in sepsis.

## Background

Sepsis and its frequent accompaniments – septic shock, systemic inflammatory response syndrome (SIRS) and adult respiratory distress syndrome (ARDS) – are major causes of multiple organ failure and mortality in hospitalized patients [1]. Overall hospital mortality rates of 21–47% have been reported among sepsis patients [2-5]. Acute renal failure (ARF) is a frequent complication [6]. Release of lipopolysaccharide endotoxin from the bacterial cell wall is among the mechanisms believed to initiate the signs, symptoms and physiologic and biochemical abnormalities characteristic of septic shock. Maldistribution of fluid in the microcirculation is typical of septic shock and results from endotoxin-induced endothelial damage. In severe sepsis, acute circulatory failure is often associated with hypovolemia and inadequate venous return, cardiac output and tissue nutrient flow [7]. Hypovolemia is a significant risk factor for mortality in sepsis [8], and these patients often require large volumes of resuscitation fluids [9]. Persistent vasodilation may also contribute to mortality among patients with sepsis [10]. Due to increased capillary permeability albumin efflux from plasma to the interstitium is increased three-fold in septic shock patients [11]. Septic patients frequently develop hypoproteinemia, which is significantly correlated with fluid retention and weight gain, development of ARDS and mortality [12].

Colloids are widely used as first-line treatment, in particular in Europe, usually in combination with crystalloids [13]. The artificial colloid hydroxyethyl starch (HES) has gained increasing acceptance for fluid management in a variety of indications. HES solutions differ according to their average molecular weight, molar substitution defined as the proportion of hydroxyethyl units substituted per glucose monomer, and substitution pattern as characterized by the ratio of substitution at the C2 and C6 positions on the glucose ring. More rapid clearance of HES molecules from plasma is observed after infusion of HES solutions with lower molar substitution, C2/C6 ratio and, to a smaller extent, molecular weight [14]. Impetus for the usage of HES has been generated by the higher unit acquisition cost of albumin [15]. Nevertheless, as previously reviewed [16], safety concerns about HES have been mounting. Some complications of HES are dose-related, and sepsis patients may require prolonged fluid administration typically with relatively high cumulative volumes. Consequently, the safety of HES in this indication needs to be appraised with particular care. The systematic review presented here is the first to assess randomized clinical trials of HES in sepsis.

## Methods

Randomized clinical trials evaluating HES in sepsis were sought by computer searches of the MEDLINE and EMBASE bibliographic databases and the Cochrane Library. Search terms included: hydroxyethyl starch; hetastarch; shock, septic; sepsis; randomized controlled trials; and random allocation. Additionally, reference lists were examined and selected specialty journals searched by hand. Eligibility was not restricted on the basis of trial endpoints, type of HES solution, time period or language of publication. Both published and unpublished trials were eligible for inclusion.

From the trial reports data were extracted on numbers of patients randomized, specific indication, fluid regimen, follow-up and endpoints. Extracted data also included the daily and cumulative HES doses and the duration of HES administration. Close attention was paid to the investigators, time periods and trial data to avoid duplication in case the same trial was the subject of multiple reports and to ensure completeness of the included evidence in the event that multiple reports of the same trial contained partially non-overlapping data.

Major findings of the included trials were qualitatively summarized and tabulated. Due to heterogeneity in the control regimens, endpoints, length of follow-up and other trial design features a quantitative meta-analysis was not judged to be feasible.

Descriptive statistics included the median and interquartile range (IQR). Calculations were performed with R version 2.2.1 statistical software (The R Foundation for Statistical Computing, Vienna, Austria).

## Results

### Included trials

**Figure 1:**
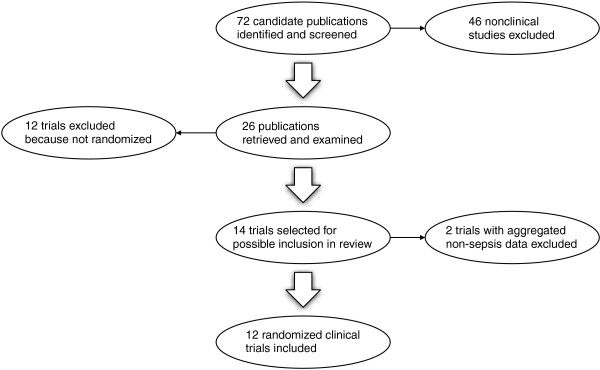
Randomized trial selection process.

The selection process for randomized clinical trials is depicted in Fig. 1. Twelve trials with a total of 1062 patients were included [7,9,17-27]. None was unpublished. With 537 patients, the recent Efficacy of Volume Substitution and Insulin Therapy in Severe Sepsis (VISEP) trial accounted for approximately half the patients in the review [27]. Two included trials were described by Rackow and co-workers in the 1980s [7,9] and 5 by Boldt et al. in the 1990s [17-22]. The remaining 5 trials conducted by various teams of investigators were reported since 2000 [23-27]. One trial in a mixed population of patients with either septic or non-septic shock was excluded because septic shock was absent in approximately one-third of the patients and study endpoint results for septic versus other forms of shock were reported only in aggregate form [28,29]. Another trial involving 27 patients with severe sepsis and 36 with postoperative SIRS was also excluded due to aggregation of data [30]. Unaggregated data for that trial were requested from the investigators, but no response was received.

### Trial characteristics

The characteristics of the included trials are summarized in Table 1. The median number of sepsis patients per trial was 30 (IQR, 26–64). Only three trials involved more than 100 patients [22,24,27]. Patients with severe sepsis or septic shock were enrolled in 6 trials [7,9,24-27]. Of the 6 other trials, 5 involved postoperative sepsis [17-22] and one sepsis with hypovolemia in ventilated and hemodynamically controlled patients [23].

**Table 1:** Characteristics of included randomized trials

**Trial**	**n**^†^	**Indication**	**Fluid Regimen**^‡^	**Follow-Up**	**Endpoints**
Falk et al., 1988 [9]	12	Septic shock	6% HES 450/0.7 or 5% albumin to 15 mm Hg target PAWP	24 h	Coagulation
Rackow et al., 1989 [7]	20	Severe sepsis and systemic hypoperfusion	10% HES 200/0.5 or 5% albumin to 15 mm Hg target PAWP or 2000 mL maximum	45 min	Cardiorespiratory function and coagulation
Boldt et al., 1995 [17,18]	30	Sepsis after major surgery	10% HES 200/0.5 or 20% albumin to 12–16 mm Hg target CVP, PCWP or both	5 days	Endothelial-related coagulation and platelet function
Boldt et al., 1996 [19]	30	Sepsis secondary to major general surgery	10% HES 200/0.5 or 20% albumin to 12–18 mm Hg target PCWP	5 days	Cardiorespiratory and circulatory variables
Boldt et al., 1996 [20]	42	Sepsis secondary to major surgery	6% HES 200/0.5, 20% albumin or pentoxifylline	5 days	Circulating soluble adhesion molecules
Boldt et al., 1996 [21]	28	Sepsis secondary to major surgery	10% HES 200/0.5 or 20% albumin to 10–15 mm Hg target PCWP	5 days	Circulatory variables
Boldt et al., 1998 [22]	150	Postoperative sepsis	10% HES 200/0.5 or 20% albumin to 12–15 mm Hg target PCWP	5 days	Hemodynamics, laboratory data and organ function
Asfar et al., 2000 [23]	34	Sepsis and hypovolemia in ventilated and hemodynamically controlled patients	500 mL 6% HES 200/0.62 or 4% succinylated modified fluid gelatin	60 min	Hemodynamics and gastric mucosal acidosis
Schortgen et al., 2001 [24]	129	Severe sepsis or septic shock	6% HES 200/0.62 up to 4 days or 80 mL·kg^-1 ^cumulative dose or 3% gelatin	34 days	ARF
Molnár et al., 2004 [25]	30	Septic shock with hypovolemia and acute lung injury	6% HES 200/0.5 or 4% modified fluid gelatin to achieve ITBVI > 900 mL·m^-2^	60 min	Hemodynamics, EVLW and oxygenation
Palumbo et al., 2006 [26]	20	Severe sepsis in mechanically ventilated patients	6% HES 130/0.4 or 20% albumin to maintain PCWP of 15–18 mm Hg	5 days	Hemodynamic and oxygenation parameters
Brunkhorst et al., 2008 [27]	537	Severe sepsis or septic shock	10% HES 200/0.5 (to 20 mL·kg^-1^·day^-1 ^limit) or Ringer's lactate to target of ≥ 8 mm Hg CVP	90 days	Morbidity and mortality

Abbreviations: ARF, acute renal failure; CVP, central venous pressure; EVLW, extravascular lung water; HES, hydroxyethyl starch; ICU, intensive care unit; ITBVI, intrathoracic blood volume index; PAWP, pulmonary arterial wedge pressure; PCWP, pulmonary capillary wedge pressure

^†^For trials with more than one indication, includes only patients with sepsis.

^‡^HES solutions specified by molecular weight/molar substitution.

HES with a molecular weight of 200 kDa and molar substitution of 0.5 (HES 200/0.5) was evaluated in 8/12 trials (67%). HES 200/0.62 was investigated in two trials and HES 130/0.4 and HES 450/0.7 in one each. The control fluid was 20% albumin in 6 trials, gelatin in 3, 5% albumin in 2 and crystalloid in one.

Ten trials (83%) were acute studies in which the observation periods ranged from less than 1 h to a maximum of 5 days. Only two trials were designed as outcome studies with follow-up of 34–90 days.

Cardiorespiratory and hemodynamic variables were endpoints of 7 trials and coagulation parameters of 3. Other evaluated endpoints consisted of extravascular lung water, gastric mucosal acidosis, circulating soluble adhesion molecules, ARF and morbidity and mortality.

### HES posology

Patients in the included trials received HES for a median of 5 days (IQR, 1–5 days). The median daily HES dose was 12.6 mL·kg^-1 ^(IQR, 11.0–13.7 mL·kg^-1^) and the median cumulative dose 49.8 mL·kg^-1 ^(IQR, 22.6–63.0 mL·kg^-1^).

### Major findings

Hemodynamic and cardiorespiratory variables were improved by HES 130/0.4 and HES 200/0.5 compared with 20% albumin [19,22,26] but not gelatin [25]. Acute Physiology and Chronic Health Evaluation (APACHE) II score was also improved by HES 130/0.4 but not 20% albumin [26]. HES 200/0.5 either improved gastric intramucosal pH (pH_i_) compared with 20% albumin [21] or avoided a decline in pH_i _observed in 20% albumin recipients [19]. On the other hand, gelatin raised pH_i _and decreased CO_2 _gastric mucosal arterial gradient, while HES 200/0.62 did not display these beneficial effects [23].

HES 450/0.7 impaired coagulation, as judged by prolonged partial thromboplastin time, and decreased platelet count [9]. These undesirable effects were not encountered in patients receiving 5% albumin. HES 200/0.5 diminished factor VIII levels compared with 5% albumin [7]. Differences in coagulation and platelet count between HES 200/0.5 and 20% albumin were not observed in one trial [22]. Compared with crystalloid, HES 200/0.5 interfered with coagulation as indicated by a higher sequential organ failure assessment (SOFA) coagulation subscore (p < 0.001) and greater median red blood cell transfusion requirement of 6 units (IQR, 4–12 units) vs. 4 units (IQR, 2–8 units) for the control group (p < 0.001) [27].

In a trial of 129 patients with severe sepsis or septic shock by Schortgen et al. [24], the groups randomized to receive HES 200/0.62 or gelatin were similar at baseline in severity of illness and serum creatinine. However, over the 34 day study observation period the incidence of ARF was increased in the HES 200/0.62 recipients (p = 0.018). In a multivariate analysis with adjustment for fluid loading before inclusion and mechanical ventilation at inclusion, HES 200/0.62 exposure was shown to be an independent risk factor for ARF (Table 2). At the conclusion of the study, ARF incidence in the HES 200/0.62 group (61%) exceeded that in the gelatin group (31%) by 30% based on Kaplan-Meier analysis. The median time to ARF among patients receiving HES 200/0.62 was 16 days. An earlier trial by Boldt and co-workers [22] failed to detect an effect of HES 200/0.5 on incidence of renal failure, possibly as a result of the short 5 day observation period. In the trial of Schortgen et al., a between-group difference in ARF incidence of only 11% was evident at the 5 day time point.

**Table 2:** HES dose administered and major findings of included randomized trials

**Trial**	**Days on HES**	**Mean mL·kg^-1 ^HES**		**Major Findings**
		*Daily*	*Cumulative*	
			
Falk et al., 1988 [9]	1	70.5^†^	70.5^†^	In HES 450/0.7 group PTT increased by 20 s (p = 0.01) and platelet count decreased by 158 × 10^3 ^mm^-3 ^(p = 0.01); no significant PTT or platelet count change in albumin group
Rackow et al., 1989 [7]	1	12.9^†^	12.9^†^	FVIII:c declined 45% in the HES 200/0.5 group compared with 5% in the albumin group (p = 0.05)
Boldt et al., 1995 [17,18]	5	8.5	42.3	Plasma thrombomodulin increased in the albumin group and remained unchanged in the HES 200/0.5 group (p < 0.05); plasma protein C among HES 200/0.5 recipients increased on days 4 and 5 without corresponding change in the albumin group (p < 0.05); maximum platelet aggregation declined in both groups (p < 0.05)
Boldt et al., 1996 [19]	5	11.0	55.2	HES 200/0.5 but not albumin increased cardiac index, RVEF, Pao_2_/Fio_2_, Do_2_I and Vo_2_I and decreased SVRI (p < 0.05 for all comparisons); pH_i _decreased in albumin but not HES 200/0.5 group (p < 0.05)
Boldt et al., 1996 [20]	5	12.7	63.7	Circulating sELAM-1 and sICAM-1 concentrations reduced by HES 200/0.5 compared with albumin (p < 0.05 for both comparisons)
Boldt et al., 1996 [21]	5	11.0	49.8	Vasopressin, endothelin-1 and norepinephrine decreased and pH_i _increased in HES 200/0.5 but not albumin group (p < 0.05 for all comparisons); ANP increased by albumin but not HES 200/0.5 (p < 0.05)
Boldt et al., 1998 [22]	5	12.5	62.4	Pao_2_/Fio_2_, Do_2_I and Vo_2_I increased and lactate decreased by HES 200/0.5 but not albumin (p < 0.05 for all comparisons); no differences in incidence of renal failure, platelet count, PT or aPTT
Asfar et al., 2000 [23]	1	7.9	7.9	Gelatin but not HES 200/0.62 increased pH_i _(p < 0.001) and decreased CO_2 _gastric mucosal arterial gradient (p < 0.0005)
Schortgen et al., 2001 [24]	4^‡^	14.0^‡^	31.0^‡^	HES 200/0.62 exposure an independent risk factor for ARF (adjusted odds ratio, 2.57; CI 1.13–5.83)
Molnár et al., 2004 [25]	1	14.3^†^	14.3^†^	No differences detected in ITBVI, EVLW or Pao_2_/Fio_2_
Palumbo et al., 2006 [26]	5	--^§^	--^§^	Target PCWP of 15–18 mm Hg maintained by both colloids; temperature, MAP, PAP, CVP, heart rate and urine output remained stable without differences between groups; HES 130/0.4, but not albumin, increased cardiac index and several oxygenation parameters (Pao_2_/Fio_2_, Do_2_I and Vo_2_I) and decreased APACHE II score (p < 0.05 for all within-group comparisons)
Brunkhorst et al., 2008 [27]	21	--^§^	70.4^¶^	Greater ARF incidence in HES 200/0.5 group (odds ratio, 1.81; CI, 1.22–2.71; p = 0.002); increased mortality at higher HES 200/0.5 doses (odds ratio, 3.08; CI, 1.78–5.37; p < 0.001)

Abbreviations: ANP, atrial natriuretic peptide; APACHE, Acute Physiology and Chronic Health Evaluation; aPTT, activated partial thromboplastin time; ARF, acute renal failure; CI, 95% confidence interval; CVP, central venous pressure; Do_2_I, oxygen delivery index; EVLW, extravascular lung water; HES, hydroxyethyl starch; FVIII:c, factor VIII coagulant activity; ITBVI, intrathoracic blood volume index; MAP, mean arterial pressure; Pao_2_/Fio_2_, ratio of partial pressure of arterial oxygen to fraction of inspired oxygen; PAP, pulmonary artery pressure; PCWP, pulmonary capillary wedge pressure; pH_i_, gastric intramucosal pH; PT, prothrombin time; PTT, partial thromboplastin time; RVEF, right ventricular ejection fraction; sELAM-1, soluble endothelial leucocyte adhesion molecule-1; sICAM-1, soluble intercellular adhesion molecule-1; SVRI, systemic vascular resistance index; Vo_2_I, oxygen consumption index

^†^Calculated from reported volume administered assuming 70 kg body weight.

^‡^Actual days on HES not specified. Maximum of 4 days imposed after start of study, and percentage of patients receiving HES longer not indicated. Daily dose stated for day 1 only. Cumulative dose reported as median.

^§^Not reported.

^¶^Median.

**Figure 2:**
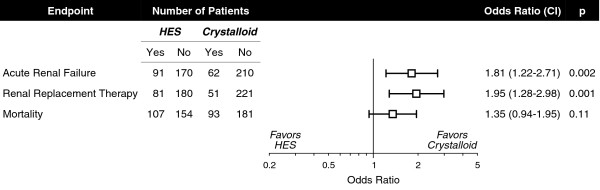
Incidence of acute renal failure, use of renal replacement therapy and mortality in patients receiving HES or crystalloid. Abbreviations: CI, 95% confidence interval; HES, hydroxyethyl starch. Based on the data of Brunkhorst et al. [27].

The recent multicenter VISEP trial assessing morbidity and mortality up to 90 days in 537 patients with severe sepsis or septic shock is the first large-scale outcome study of HES 200/0.5 in any clinical indication [27]. The HES 200/0.5 and crystalloid groups were well-matched at baseline in severity of illness and serum creatinine. HES 200/0.5 infusion compromised renal function (p = 0.02) as reflected by a higher SOFA renal subscore than that of the control group. The incidence of ARF and use of renal replacement therapy (RRT) were both increased by HES 200/0.5 (Fig. 2). RRT usage was positively correlated with cumulative HES 200/0.5 dose (p < 0.001). Even in the subset of patients receiving exclusively lower HES 200/0.5 doses (≤ 22 mL·kg^-1^), higher ARF incidence (p = 0.04) and RRT utilization (p = 0.03) were demonstrated in comparison with the crystalloid control group.

In the VISEP trial there was also an overall trend toward increased mortality among HES 200/0.5 recipients (Fig. 2). Mortality at 90 days was correlated with cumulative HES 200/0.5 dose (p = 0.001) and significantly increased (p < 0.001) in patients receiving > 22 mL·kg^-1 ^HES 200/0.5 (58%) for at least one day than lower doses (31%).

## Discussion

Until relatively recently, randomized trial evidence concerning HES for fluid management in sepsis patients has stemmed almost entirely from small acute studies, often focused on cardiorespiratory and hemodynamic endpoints. These trials were thus not designed to evaluate safety or outcomes, and renal function in particular was not evaluated. The report of Schortgen et al. [24] was to first to raise serious concern that HES might adversely affect renal function in sepsis. The results of that trial should perhaps have been unsurprising in light of earlier randomized trials indicating deleterious effects of HES on the kidney in cardiac [31] and abdominal surgery [32] and renal transplantation [33]. The VISEP trial [27] has now furnished convincing confirmation that HES increases ARF incidence in sepsis.

The administration of HES 200/0.5 in the VISEP trial has also put to rest the argument that the adverse renal effects observed by Schortgen et al. might have been due to their use of the more highly substituted HES 200/0.62 solution. On the other hand it should be recognized that the results of sepsis trials involving repeated HES infusion over a period of several days or more cannot necessarily be extrapolated to other settings such as postoperative fluid management involving lower HES doses for a shorter time.

The VISEP trial has also provided the first evidence that HES may increase mortality among sepsis patients. A trend toward higher mortality was observed among all recipients of HES compared with crystalloid, and mortality was significantly increased by higher HES doses. These data are in contrast to the results of the SAFE trial [34] comparing 4% albumin with normal saline. In the subset of 1218 SAFE trial patients with severe sepsis, a trend toward reduced mortality was evident in the albumin group (odds ratio, 0.81; 95% confidence interval, 0.63–1.04; p = 0.09). In light of these disparate survival trends, a need exists for an adequately powered outcome trial directly comparing HES and albumin in sepsis.

Schortgen et al. detected no effect of HES 200/0.62 on survival; however, the duration of follow-up in their trial was 34 days. In the VISEP trial the Kaplan-Meier survival curves for the two randomized groups began to diverge only at approximately 30 days and were clearly separated thereafter. It is thus possible that in the trial of Schortgen et al. a mortality difference might have become apparent with longer follow-up.

The mechanisms that might account for undesirable HES effects on kidney function and possibly survival in sepsis are not understood. One putative mechanism is renal ischemia [24]. HES has been shown to increase plasma viscosity *in vitro *compared with albumin [35]. In a rat model of severe hemorrhagic shock, both 6% HES and 5% albumin restored macrocirculatory function as measured by mean arterial pressure [36]. However, only albumin completely returned mesenteric microcirculatory blood flow to the baseline level. Furthermore, albumin was effective in restoring mesenteric lymphatic output, while HES was not (p < 0.05).

Since more than 30 years ago there has been evidence that HES might impair reticuloendothelial system (RES) function, thereby impairing host defenses against sepsis and possibly contributing to multiple organ failure, including ARF, and mortality [37]. A substantial proportion of administered HES cannot be metabolized acutely and undergoes uptake and storage by the RES, most notably in macrophages including those localized in the kidney [38-42]. In a quantitative necropsy specimen study of 12 young adult patients who had died due to sepsis and multi-organ failure after receiving a mean of 258 mL·day^-1 ^HES 200/0.5, the highest mean major organ HES tissue concentration was measured in the kidney (13.7 mg·g^-1^) [43]. The effects of plasma substitutes on RES function in mice were investigated by intraperitoneal injection of *Salmonella enteritidis *endotoxin [37]. Host defenses against this endotoxin are mediated by RES macrophages. Prior infusion of HES but not plasma increased the lethality of endotoxin injected either 1 h (p < 0.05) or 3 h (p < 0.01) subsequently. Similarly, in a murine hemorrhagic shock model no HES-resuscitated animal survived intraperitoneal injection of live *E. coli *at 1 h after resuscitation, whereas survival with shed blood resuscitation was 64% [44]. In contrast to these two studies involving acute septic challenge within 1–3 h after HES administration, a delayed challenge in the form of cecal ligation and puncture 48 h after HES infusion did not increase mortality in rats [45]. In any case, there is at present no clinical evidence indicating HES-mediated impairment of RES function in sepsis, and clinical studies will be required to delineate the role, if any, of the RES in explaining the observed deleterious effects of HES among septic patients.

A variety of HES solutions are available that differ in average molecular weight, molar substitution, C2/C6 ratio and solvent. It has often been claimed that a particular HES solution may be devoid of safety problems displayed by others. Several recent evidence-based reviews have challenged this contention [16,46-48]. Similar types of complications, including impaired kidney function [48], have been encountered clinically across the entire spectrum of HES solutions. These adverse effects appear to reflect the intrinsic pharmacologic properties of the HES molecule rather than differences between individual HES solutions [49]. For instance, HES 130/0.4 was shown to impair renal function assessed by four sensitive markers in a randomized trial of elderly cardiac surgery patients [50]. HES 450/0.7 in Ringer's lactate vehicle was independently associated with reduced glomerular filtration rate in a retrospective study of 238 consecutive coronary artery bypass graft patients [51]. The safety of either solution for fluid management in sepsis would need to be demonstrated in clinical trials.

## Conclusion

Compelling evidence is now at hand indicating that HES infusion places sepsis patients at increased risk for ARF. New data also suggest the possibility of poorer survival among sepsis patients receiving HES, especially higher doses. Clearly, HES 200/0.5 and HES 200/0.62 cannot now be recommended in sepsis. The effectiveness and safety of other HES solutions in this indication remain to be determined in future clinical trials.

## List of abbreviations

ARDS: Adult respiratory distress syndrome; ARF: Acute renal failure; HES: Hydroxyethyl starch; IQR: Interquartile range; pH_i_: Gastric intramucosal pH; RES: Reticuloendothelial system; RRT: Renal replacement therapy; SIRS: Systemic inflammatory response syndrome.

## Competing interests

The author(s) declare that they have no competing interests.

## Authors' contributions

CJW is sole author.

## Pre-publication history

The pre-publication history for this paper can be accessed here:


